# “Time is of the essence”: relationship between hospital staff perceptions of time, safety attitudes and staff wellbeing

**DOI:** 10.1186/s12913-021-07275-6

**Published:** 2021-11-20

**Authors:** Louise A. Ellis, Yvonne Tran, Chiara Pomare, Janet C. Long, Kate Churruca, Zeyad Mahmoud, Winston Liauw, Jeffrey Braithwaite

**Affiliations:** 1 Centre for Healthcare Resilience and Implementation Science, Australian Institute of Health Innovation, Macquarie University, Level 6, 75 Talavera Road, NSW Sydney, Australia; 2 Université de Nantes, LEMNA, F-44000 Nantes, France; 3 South Eastern Sydney Local Health District, Sydney, Australia

**Keywords:** Temporality, Time, Job satisfaction, Burnout, Hospital

## Abstract

**Background:**

Hospitals are perceived as fast-paced and complex environments in which a missed or incorrect diagnosis or misread chart has the potential to lead to patient harm. However, to date, limited attention has been paid to studying how hospital sociotemporal norms may be associated with staff wellbeing or patient safety. The aim of this study was to use novel network analysis, in conjunction with well-established statistical methods, to investigate and untangle the complex interplay of relationships between hospital staff perceived sociotemporal structures, staff safety attitudes and work-related well-being.

**Method:**

Cross-sectional survey data of hospital staff (*n *= 314) was collected from four major hospitals in Australia. The survey included subscales from the Organizational Temporality Scale (OTS), two previously established scales of safety attitudes (teamwork climate and safety climate) and measures of staff-related wellbeing (job satisfaction, emotional exhaustion, depersonalisation).

**Results:**

Using confirmatory factor analysis, we first tested a 19-item version of the OTS for use in future studies of hospital temporality (the OTS-H). Novel psychological network analysis techniques were then employed, which identified that “pace” (the tempo or rate of hospital activity) occupies the central position in understanding the complex relationship between temporality, safety attitudes and staff wellbeing. Using a path analysis approach, serial mediation further identified that pace has an indirect relationship with safety attitudes through wellbeing factors, that is, pace impacts on staff wellbeing, which in turn affects hospital safety attitudes.

**Conclusions:**

The findings of this study are important in revealing that staff wellbeing and safety attitudes can be significantly improved by placing more focus on temporal norms, and in particular hospital pace. There are implications for increasing levels of trust and providing staff with opportunities to exercise greater levels of control over their work.

**Supplementary Information:**

The online version contains supplementary material available at 10.1186/s12913-021-07275-6.

## Background

Hospitals are fast-paced, complex, and exigent workplaces characterised for example by the quick turn over of patients and the rapid response of emergency teams [1]. Hospital clinical staff experience day-to-day work with deadlines, schedules and routines that are unlike any other profession in the modern world [2]. Faced with ever-increasing cost constraints as well as demands for services, hospital staff, more than ever before, are experiencing accelerating workloads and pressures to work quickly and efficiently [1]. Hospitals are increasingly being controlled [3, 4] and organised by chronological time based targets, such as the “four-hour rule” in the United Kingdom and Australia by which patients must be seen, assessed and transferred [5, 6] or discharged within four hours of presentation to the emergency department [3, 4]. Questions have been raised as to whether the accelerating workloads of hospital staff are sustainable, and whether a faster pace threatens the quality of care delivered [1, 7, 8].

As hospital staff face pressures to deliver care at a faster pace there are also potential negative implications for staff wellbeing (e.g., burnout, job satisfaction) and patient safety [9, 10]. Workload and time pressure have been identified as a major contributor to staff burnout [11, 12]. For example, as demand for acute care services has increased, hospital staff experience pressure to work at a faster pace to deliver care to patients in need [1, 13]. Working at levels of excessive effort for a period without much recovery time has been identified as a precursor to burnout [11], and burnout has been shown to affect the quality of care provided to patients [9, 14, 15].

### Temporality research and its applicability to healthcare

Staff experiences of time—often referred to as “temporality” in the academic literature—have been examined in a range of organisational settings, but to date have received little attention in healthcare [16]. In previous workplace temporality studies, researchers have observed that time does not merely exist objectively as clock time, but is also experienced in subjective, non-linear ways [17], with an individual’s experience of time being associated with organisational events rather than identifiable time periods [18]. The experience of time also has a social component; in the workplace, time is something that is constructed through individual interactions with those around them [2]. From this perspective, “time is taken to mean shared experiences of time, personal conceptions of time, as well as institutionally driven, formal temporal parameters on members work processes” ([19], p. 319). A review of workplace temporality research identified that organisational units and their members create *sociotemporal norms* (i.e., shared beliefs about temporality) through regularised patterns of interaction [17, 20]. These shared beliefs are reflected through their *enactments* of time (i.e., the way employees perform time-based activities and include the dimensions of pace, punctuality, scheduling, linearity, flexibility). Ballard and Seibold (2006) developed a multidimensional instrument, the Organizational Temporality Scale (OTS), to measure how organisational group members “perform” time; this measure was developed and tested with staff and student members (*n *= 395) of a university in the United States of America. Further research within the same university identified that individuals who perceived their work as more flexible reported higher job satisfaction, while those who perceived work as fast paced were less satisfied [19].

Whilst previous research has made gains in understanding how cultural attitudes and perspectives about time within hospitals affect outcomes of care, little research has investigated how temporal enactments in hospitals are associated with staff wellbeing and patient outcomes. Utilising five scales from the OTS, one previous study examined the impact of hospital employees perceived temporal enactments (pace, punctuality, scheduling, linearity, flexibility) on their perceptions of urgency and their perceived ability to segregate their work and home life [2]. Results of this study revealed that scheduling and linearity were positively correlated predictors of work-life interference, broadening our understanding of the work-life interference levels hospital employees experience on a daily basis. However, to date, the OTS has not been validated in a hospital setting, and further research is needed to investigate the impact of hospital temporality on staff wellbeing and patient safety.

### Aims of the present study

The primary purpose of the present study was to empirically examine the relationship between hospital staff’s enactments of time, safety attitudes, and two key indicators of work-related wellbeing: burnout and job satisfaction. Our study had four specific aims:

1. *To validate a survey instrument for measuring temporal enactments in hospital settings using confirmatory factor analysis (CFA).*
2. *To investigate the extent to which staff demographical profiles have an impact on staff temporal enactments at work.* Here we focused on occupational role and years of organisational experience, as two key demographic variables previously hypothesised to have a potential impact on hospital employee’s temporal enactments at work [2], as well as age differences.
3. *To examine the complex interconnected relationships between temporal enactments, safety attitudes and work-related wellbeing in a hospital system using a psychological network.* A psychological network [21] is an emerging network analytic method based on graph theory, with nodes representing observed variables, connected by “edges” (i.e., the links between nodes) representing statistical relationships. One specific advantage of the network approach is that it defines observed variables as constituents of a complex system of direct interactions, often resulting in unique and important contributions to our knowledge about the relationships amongst these constructs [22].
4. *To demonstrate the relationships observed from the pathways in the psychological network with mediation models using a path analysis approach.*

## Method

### Participants and setting

The sample in this study was part of a larger project examining organisational culture and care delivery at four public hospitals in metropolitan Sydney, Australia, of which all are administered by the New South Wales Department of Health. The four hospitals were of similar size (all above 500 beds), and varying geographic locations and socio-economic disadvantage across greater metropolitan Sydney [23]. Each of the participating hospitals offered similar types of services (e.g., emergency department, intensive care, surgical, aged care). Hospital staff working at the four hospitals were invited to participate in the study through an e-invitation sent through their work email address. The email was distributed by hospital administration via their staff distribution lists which provided a link to an online version of the survey using *Qualtrics* software [24]. The ethical conduct of the study was approved by South Eastern Sydney Local Health District (Ref No. 16/363). Participants provided written informed consent and understood that their participation was voluntary and anonymous, with no incentives offered to enhance enrolment. To apply the statistical analysis in this study, 5 to 10 observations per estimated parameter [25] and a minimum sample size of 100 to 200 [26] were required.

### Survey

#### Perceived temporal enactments

The current study used subscales from the OTS, created by Ballard and Seibold [27]. This scale was designed to measure how organisational members experience enactments of time. For the purpose of this study, six subscales (flexibility, linearity, pace, scheduling, punctuality and delay) including a total of 21 items were used to measure staff members’ temporal enactments (see Additional file 1). The items were prefaced with the following statement: “Think about the way you and your co-workers refer to time in the course of carrying out your daily tasks at work. Please rate each of the following words or phrases based on how well they describe the way you and others in your immediate work group or unit talk about time”. The first subscale, flexibility, assesses the degree of rigidity in time-organising and task-completion strategies [27] and included items such as “set in stone” and “rigid”. Pace refers to tempo or rate of activity [27] (e.g., “hurried” and “rapid”. Linearity is associated with actual task execution, and is characterised by doing one thing at a time [27] (e.g., “carried out step by step”, “having a specific order”). Scheduling is a dimension concerned with the extent to which plans, activities and events are formalised [27] and includes items such as “tightly scheduled” and “unplanned”. The final subscales, punctuality (e.g., “prompt”) and delay (e.g., “running late”), refer to the preciseness of timing. Ballard and Seibold (2004) conceptualised punctuality and delay as separate constructs “because of the multiple temporal commitments inherent in workplace responsibilities and job roles, and because of norms surrounding timing” (p. 6). For example, although a specific project may be running behind schedule, organisation members may still respond to work requests quite promptly [20]. All of the items were assessed on a six-point Likert scale (1=strongly disagree to 6= strongly agree). An examination of the psychometric properties of these items are presented in the Results section and pertain to Aim 1 of the current study.

#### Safety attitudes

Safety attitudes was measured using the Safety Attitudes Questionnaire (SAQ) [28]. The SAQ is a validated instrument used to measure attitudes and perceptions in various safety-related domains in healthcare. Previous research has demonstrated a significant association between SAQ scores and patient outcomes [29]. Two subscales were included: teamwork climate (six items) and safety climate (six items). Questions were measured on a five-point Likert-type scale (1=strongly disagree to 5=strongly agree). In the present study, we found acceptable internal consistency reliabilities for the two SAQ subscales for teamwork climate (Cronbach’s *α =* 0.84) and for safety climate (Cronbach’s *α =* 0.80), similar to that reported by Sexton, Helmreich [28] (Raykov’s ñ=0.90).

#### Work-related wellbeing

Job satisfaction and burnout were used as indicators of work-related wellbeing. Job satisfaction was assessed using three items from the Job Diagnostic Survey (JDS) [30]. Responses were rated on a five-point Likert scale (1=strongly disagree to 5=strongly agree). In the present study, we found acceptable internal consistency for the scale (Cronbach’s *α =* 0.88), consistent with that reported by Bowling and Hammond [30] (Cronbach’s alpha=0.84).

Burnout was measured using a 10-item version of the Maslach Burnout Inventory (MBI [31–33]. Due to the inappropriateness of the third subscale, personal accomplishment, for use in healthcare settings [34, 35] only two subscales of burnout—emotional exhaustion (five items) and depersonalisation (five items)—were used. Items were measured on a seven-point Likert scale (1=strongly disagree to 7= strongly agree). In the present study, the internal consistency coefficients for emotional exhaustion (Cronbach’s *α =* 0.93) and depersonalisation (Cronbach’s *α =* 0.91) were excellent (Cronbach’s *α =* 0.93).

### Data analysis

Participants missing more than 10 % of survey data were excluded. Remaining missing values were imputed using the Expectation Maximisation (EM) Algorithm within SPSS v25 [36].

To address Aim 1, the 21 items from the OTS assessing temporal enactments were evaluated psychometrically via CFA. Each item was loaded on the one factor it purported to represent. Goodness-of-fit was assessed using the Tucker Lewis Index (TLI), Comparative Fit Index (CFI), Root Mean Square Error of Approximation (RMSEA) and the relative Chi-square (chi-square/df). The TLI and CFI yield values ranging from zero to 1.00, with values greater than 0.90 and 0.95 being indicative of acceptable and excellent fit to the data [37]. For RMSEA, values less than 0.05 indicate good fit, and values as high as 0.08 represent reasonable errors of approximation in the population [38]. Chi-square tests are sensitive to sample size [39], therefore the relative chi-square (chi-square/df) was used as an index of fit, with values less than two indicating a good model fit [40]. Reliability of each of the subscales was assessed through Cronbach’s alpha (using SPSS v25) and composite reliability (using AMOS v25).

To address Aim 2, separate one way ANOVAs were used to examine whether occupation, experience or age had a significant effect on perceived temporal enactments. Analyses were conducted in SPSS and a significance value of 0.05 was used.

For Aim 3, psychological network analysis estimated from a network of partial correlation coefficients was used to visualise the relationship between factors for temporal enactments (flexibility, linearity, pace, scheduling, punctuality and delay), safety attitudes (teamwork climate, safety climate) and work-related wellbeing (job satisfaction, emotional exhaustion, depersonalisation). The R package qgraph was used to estimate the network [21]. A correlation matrix was computed from the 11 observed variables measuring temporal enactments, safety attitudes and work-related wellbeing. An undirected weighted network was constructed with the correlation matrix. Edges from the network depict partial correlations between each pair of nodes after controlling statistically for all other variables. Using this method, 100 different network models were estimated with different degrees of sparsity and the model with the lowest information criterion was selected. The model computes regularised partial correlations between pairs of nodes, eliminates spurious connections from the influence of other nodes within the network, and shrinks trivial and small associations to zero. In addition, we applied a precision matrix threshold to ensure high specificity for the pathways. Network density was calculated to measure the percentage of connections over the total number of possible connections.

In addition to the visualization of networks, we also examined centrality indices; network metrics used to identify the relative importance of nodes in the structure of the network. Three centrality indices were examined: “betweenness” measuring the number of times a node acts as a bridge along the shortest path between any other pair of nodes; “closeness” measuring the average distance of a node from all other nodes in the network; and “strength” measuring the sum of the edge weights attached to each node (or the number of connections) [41]. Each of the centrality indices were standardized and values greater than zero reflected greater centrality in the network.

To address Aim 4, path analyses were performed using the PROCESS macro (model 4) V3.4 in SPSS to estimate the mediation models. Non-parametric bootstrapping analyses were used to test the models in this study. Mediation was found to be significant if the 95 % bias corrected confidence intervals for the indirect effects do not include zero. Common method bias was checked with the variance inflation factor (VIF).

Finally, because the data were collected from a single source, there is a risk that common method bias may jeopardise the interpretation of the results. In order to assess this bias, we employed the Harman single-factor approach [42] and full collinearity assessment approach [43]. The single un-rotated factor with all survey items entered explained less than 50 % of the variance (27.6 %), thus providing evidence that common method bias was not a pervasive problem in this study. Further, across the path analysis model, VIF values ranged from 1.094 to 1.557, which is lower than the common method bias cut-off of 3.3.

## Results

### Descriptive statistics

Participants were 415 staff from four hospitals in Australia. After excluding participants with more than 10 % of missing data in the survey, the remaining sample was reduced to 314. Of the 314 participants, most were female (75.6 %), worked as a nurse (38.5 %) or doctor (19.3 %), and had been working in the same hospital for three or more years (75.1 %). The characteristics of the survey respondents are presented in Table 1. Descriptive statistics for all items are presented in Additional file 1.

**Table 1:** Characteristics of survey respondents (*N *= 314)

		*N*	*%*
Sex	Male	66	24.4
	Female	204	75.6
Age	18-24 years	7	2.5
	25-34 years	72	26.1
	35-44 years	66	23.9
	45-54 years	67	24.3
	> 55 years	64	23.2
Years at hospital	< 1 year	36	13.3
	1-2 years	31	11.5
	3-5 years	61	22.6
	6-10 years	58	21.5
	> 11 years	84	31.1
Occupation	Administrative staff	28	10.2
	Allied health professional	44	16.0
	Management	14	5.1
	Physician/Medical officer	53	19.3
	Registered or enrolled nurse	106	38.5
	Other	30	10.9

*Note*: Responses may not equal 314 responses due to missing data

### Aim 1: Validation of survey instrument for measuring temporal enactments in hospital settings

The first aim was to validate a survey instrument for measuring sociotemporal norms in hospital settings. The 21 item six-factor model of temporal enactments produced an inadequate fit to the data, χ^2^ (174)=388.37, TLI=0.944, CFI=0.954, RMSEA=0.063, and a relative chi-square value of 2.23. Inspection of the standardised factor loadings and modification indices for three items (FASTPACE, ONEATIM and ONTIME) suggested that their removal may improve model fit. The removal of these three items resulted in an improved and satisfactory model fit, χ2 (120)=238.60, TLI=0.963, CFI=0.971, RMSEA=0.056, and a relative chi-square value of 1.99. The standardised factor loadings for the remaining 18 items ranged from 0.75 to 0.95. The retained items are presented in Table 2, along with their factor loadings. Cronbach’s alphas for the final items are also shown in Table 2, showing that all six scales demonstrated acceptable levels of reliability.

**Table 2:** CFA and reliability results for reduced 19 item measure of temporal enactments

Construct	Item	Factor loadings	Coefficient alpha	Composite reliability
Flexibility	SETSTONE	.85	.94	.94
	RIGID	.95		
	FIXED	.87		
	INFLEXIB	.75		
Pace	HURRIED	.89	.94	.94
	RAPID	.93		
	QUICK	.87		
	RACING	.88		
Linearity	STRUCTUR	.81	.88	.88
	HAVORDER	.88		
	STPBYSTP	.83		
Scheduling	UNSCHED	.73	.82	.83
	UNPLAN	.95		
Delay	BEHNDSCH	.84	.92	.92
	RUNLATE	.95		
	DELAYED	.88		
Punctuality	PUNCTUAL	.87	.87	.87
	PROMPT	.89		

### Aim 2: Staff demographics and temporal enactments

The second research aim was to test whether employee demographical profiles had an impact on hospital employee’s sociotemporal norms at work. Separate one-way ANOVAs for occupation revealed that nurses reported significantly higher levels of pace (F(1,273)=4.83, *p *<.05) and delay (F(1,273)=4.50, *p *<.05), but significantly lower levels of linearity (F(1,273)=6.95, *p *<.01), compared with other occupations. There were no significant effects of years of organisational experience or age on any temporality subscales.

### Aim 3: Psychological Network

**Fig. 1:**
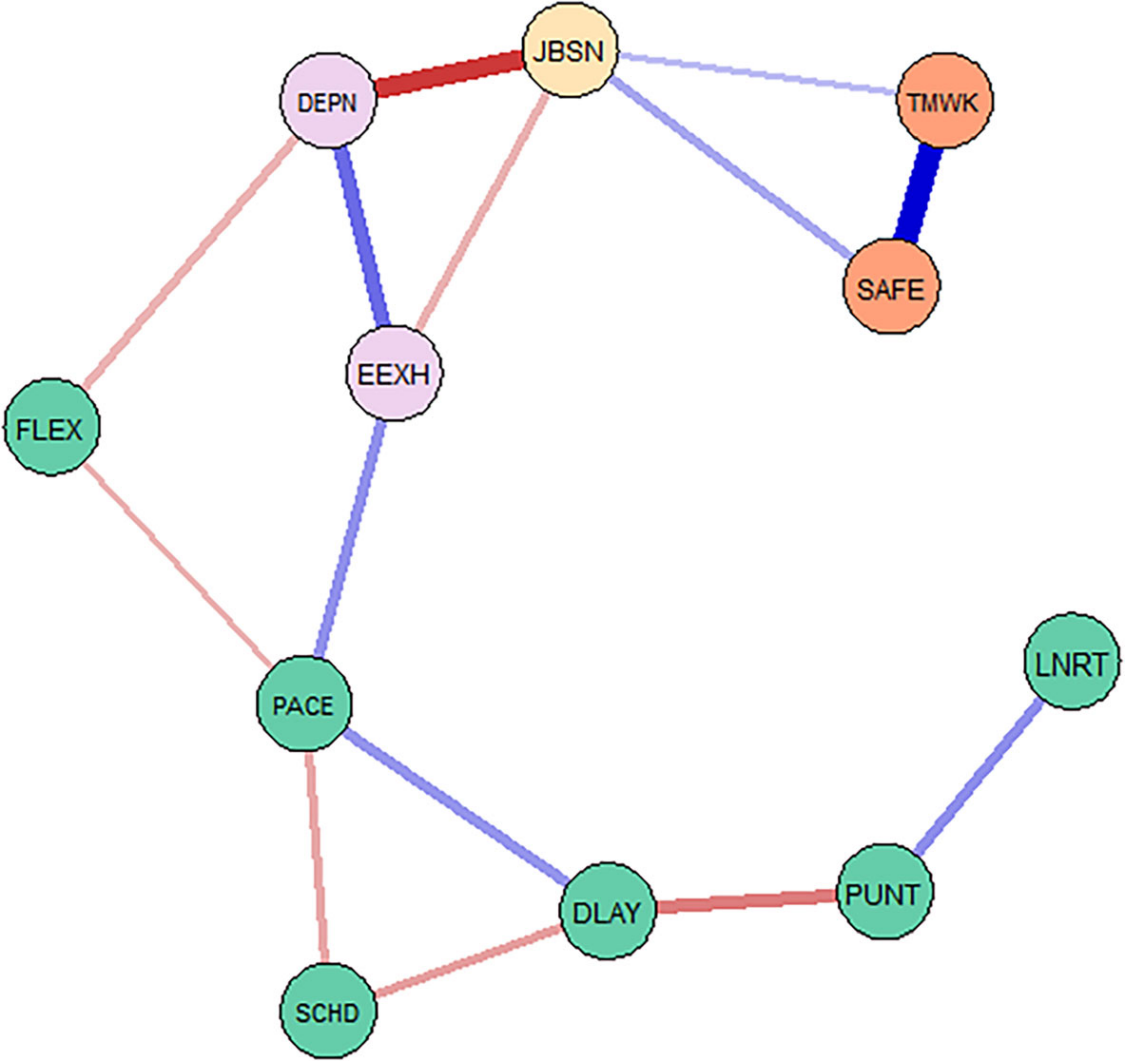
Psychological network depicting temporality, wellbeing and culture scales. Note. The nodes (circles) represent each of the 11 measured scales. The edges (lines) reflect the magnitude of the association between the nodes with thicker edges representing stronger relationships. Positive relationships are depicted as blue edges and negative associations are depicted as red edges. Each survey tool is represented by a different node colour. Temporality (green): FLEX=flexibility, PACE=pace, LNRT=linearity, SCHD=scheduling, DLAY=delay, PUNT=punctuality; Burnout (purple): EEXH=emotional exhaustion, DEPN=depersonalisation; Job satisfaction (yellow): JBSN=job satisfaction; Safety attitudes (orange): TMWK=teamwork climate, SAFE=safety climate

Figure 1 shows the resultant network structure of factors from temporal enactments, with safety attitudes and staff wellbeing factors. With a high precision matrix threshold applied, there were 15 connections between nodes and network density of 27 %. As seen in Fig. 1, pace can be regarded as the bridge between four of the temporal enactments (linearity, punctuality, delay, and scheduling) and the staff wellbeing and safety attitude factors; that is, in order to examine the relationships between these variables with wellbeing and safety, they must pass through the pace node first. It is important to note here that although pace is central, serving as a bridge between four of the other temporal enactments and the wellbeing and cultural scales, it doesn’t necessarily mean they don’t have explanatory power, but from the network pace appears to mediate these pathways between them. However, pace was not a bridge for flexibility. As shown in Fig. 1, flexibility was negatively associated with pace [note in Figs. 1 and 3, positive associations are depicted as blue edges (i.e., lines) and negative associations are depicted as red edges].; that is less flexibility is related to a faster pace.

**Fig. 2:**
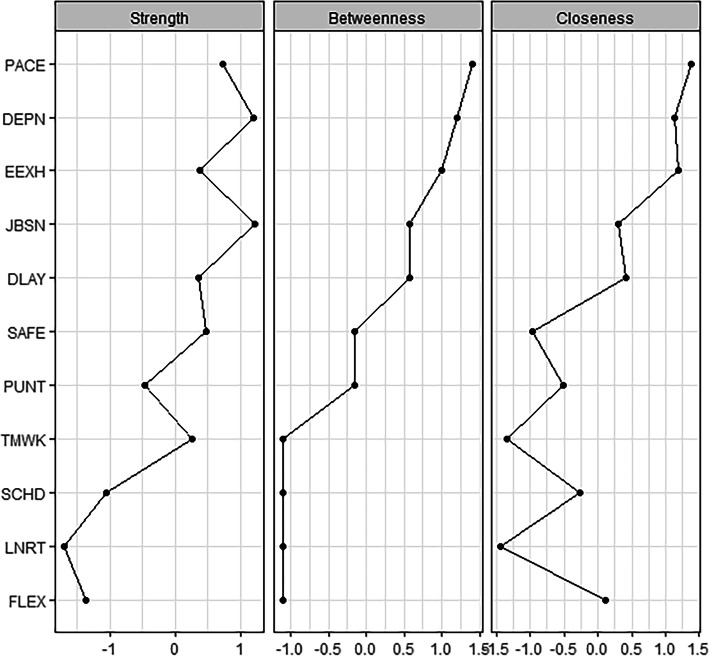
Centrality plot for the concentration network depicting the betweenness, closeness and strength for each scale. Note. FLEX=flexibility, PACE=pace, LNRT=linearity, SCHD=scheduling, DLAY=delay, PUNT=punctuality, EEXH=emotional exhaustion, DEPN=depersonalisation, JBSN=job satisfaction, TMWK=teamwork climate, SAFE=safety climate

Figure 2 shows the result of the centrality analyses for each of the 11 variables. Node centrality analyses identified “PACE” as the node exerting the strongest influence within the entire network. Pace is shown to have the highest betweenness and closeness centrality and also a high strength index.

**Fig. 3:**
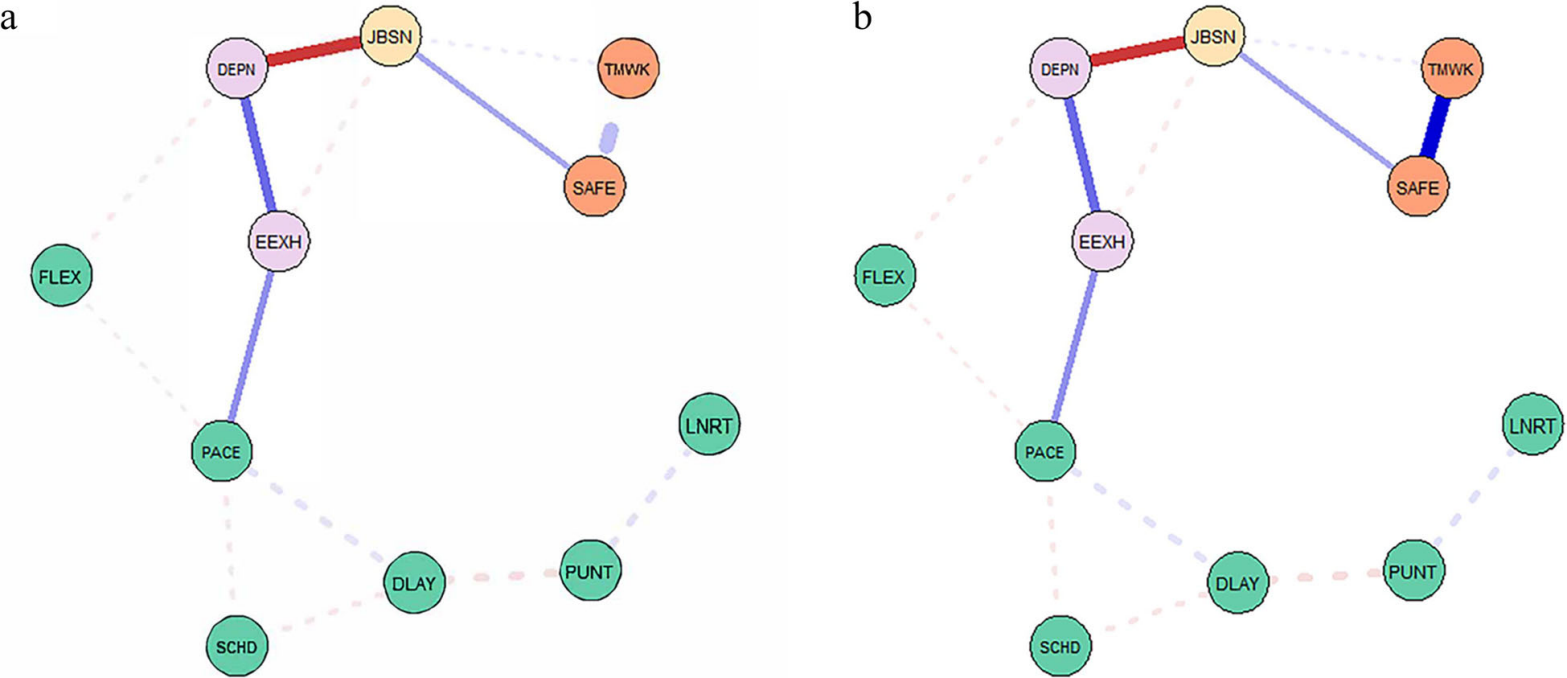
**a** and **b** Shortest paths between PACE and cultural factors. Note. FLEX=flexibility, PACE=pace, LNRT=linearity, SCHD=scheduling, DLAY=delay, PUNT=punctuality, EEXH=emotional exhaustion, DEPN=depersonalisation, JBSN=job satisfaction, TMWK=teamwork climate SAFE=safety climate

We then examined the shortest paths between pace and safety attitude factors (teamwork climate, safety climate). Figure 3a shows the network depicting the relationship between pace and teamwork as passing through burnout factors (EEXH, DEPN) and then job satisfaction, and safety climate before reaching teamwork. Figure 3b. shows that for safety climate, pace passes through burnout factors (EEXH, DEPN), then job satisfaction before reaching safety climate.

### Aim 4. Mediation models using path analysis

The paths identified from the network analysis suggested the interconnected relationships between temporality, work-related wellbeing and safety attitudes in the hospital system. From this, a serial mediation path models were generated to demonstrate the explanatory pathways for the relationship between pace and safety attitudes. For the explanatory model for pace with safety attitudes, the network analysis suggested two serial wellbeing mediators—burnout and job satisfaction. Results for the path model were based on 5000 bootstrapped samples.

**Fig. 4:**
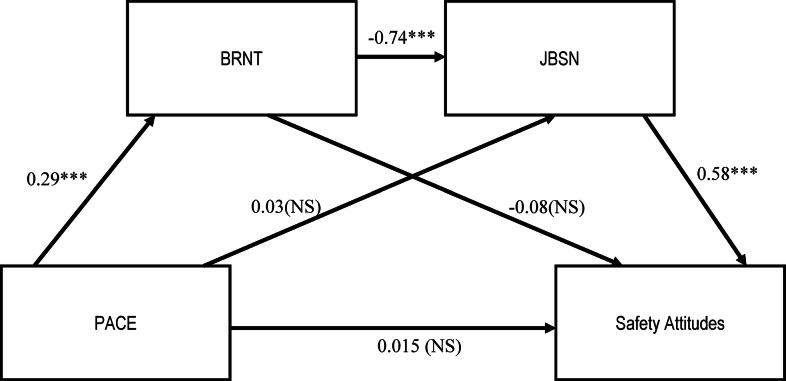
Mediation pathway for pace with safety attitudes with standardised beta values. Note. * *p* < .05, ** *p* < .01, *** p < .001

For pace with the overall safety attitudes composite, the results showed a significant total effect (β= -0.12, SE=0.03, *p *<.05). The serial indirect pathway (pace>burnout>job satisfaction>safety attitudes) was found to be significant (Boot lower CI = -0.19 and Boot higher CI = -0.07) but the direct effect between pace and safety climate was not significant (Bdirect=0.007, SE=0.02, *p *=.75) indicating a full mediation. The wellbeing mediator, burnout and job satisfaction, fully mediated the relationship between pace and safety attitudes, indicating that this relationship can also be explained through a mediational process (see Fig. 4).

## Discussion

Hospitals are highly complex environments with unique temporal commitments and responsibilities. As identified by Barrett (2014), in a hospital environment, “one can imagine that a group’s preoccupation with deadlines and task completion is a common temporal norm, as the recurring cycles of emergency situations can cause hospital staff to feel as though they are always running out of time” (p.450). This study used novel network analysis techniques, in conjunction with well-established methods (CFA and path analysis), to empirically examine the complex relationships between staff perceptions of time, safety attitudes and work-related wellbeing.

In recent years, measures of safety culture, burnout and job satisfaction have proliferated [44]. They are now considered central to the understanding of patient safety and are increasingly being used as part of system-based approaches to reduce errors in healthcare settings [45]. However, to our knowledge, few measures of temporality currently exist, none have been validated in a hospital setting, and only one previous study [2] has explored the relationship between sociotemporal norms in hospitals and staff wellbeing, despite its importance. Hence, this study is the first to test and validate the OTS for use in hospital settings. The tested 19-item version of the survey (OTS-H) can be used to further our understanding of the impact of hospital temporality on staff wellbeing and patient safety.

The second aim was to explore how staff demographics impact the temporal enactments of hospital employees. Since occupation has previously been proposed to be a relevant distinction among organisational groups and their experiences of time outside of healthcare [46, 47], it was not surprising that we found this variable to have a significant impact on hospital staff perceptions of temporality. Specifically, we identified that nurses reported significantly higher levels of pace and delay, but significantly lower levels of linearity, compared with other occupations. Indeed, previous research has suggested that nurses worldwide are experiencing increased time pressure, as a result of having multiple roles to fulfil, which is being further exacerbated by a global nursing shortage [48]. Further, reports of insufficient time for completing required tasks are common among nurses[49].

Third, we used a novel network analytic approach to examine the interplay and pathways between temporality, safety attitudes and work-related wellbeing. The results of this analysis identified that, with the exception of flexibility, pace can be regarded as the bridge between temporal actors and staff wellbeing and hospital culture factors. In other words, pace occupies the central position in the network and plays a vital role, having a negative effect on staff wellbeing and safety attitudes. A serial mediation model further revealed that pace has an indirect relationship with safety attitudes through wellbeing factors, that is, pace affects staff wellbeing, which in turns influences hospital safety attitudes. This is consistent with previous research findings examining psychological wellbeing of hospital staff. In a study of operating theatres [10], clinical staff reported that the lack of control over the pace of work was a key contributing factor to their experiences of powerlessness and alienation, especially when coupled with a high degree of unpredictability. The inability to adjust efforts in accordance with energy and skills levels was described as source of stress and strain as staff were unable to regulate the pressure exerted on them in their work situations.

In this context pace should not be confused with workload (i.e., the amount of work to be done), although there is considerable overlap between the two: people with a high workload often move quickly [50, 51]. Levine (2005) further suggested that when someone says they are busy, they may be experiencing high levels of either pace or workload, or both. We are also not suggesting that emphasis should be placed on reducing hospital busyness [50]. Rather, we hold the view that there may be an *optimal* level of busyness for hospitals to function well: “too fast and mistakes are made, staff leave exhausted and burnt out, and patients are dissatisfied with their care; but too slow and things do not get done at day’s end, boredom settles in, expenses escalate, and wait lists blow out” (p. 2) [1]. Related research outside of healthcare, has suggested that optimal levels of busyness may even have positive benefits: it can be energising, produces elation, and increases perceived productivity [52].

This research shows that our understanding of safety attitudes and work-related wellbeing can be significantly improved by placing more focus on temporality in hospitals, and in particular pace. This has multiple implications for healthcare managers and policy makers concerned with staff wellbeing and safety and quality in healthcare. The results highlight the central role pace plays on staff wellbeing and safety attitudes, and by extension on the safety and quality of care delivered to patients. These findings call for targeted managerial interventions and organisational policies aimed at giving staff more control over the pace of their work by moving away from overly rigid time-based targets, such as the “four-hour rule”. It is indeed crucial for health workers to be able to vary the rhythm at which different activities are executed to be in line with their skills and overall demands placed on them. Such policies and interventions could for instance ensure compliance with adequate staffing and training levels which could lead to a better division of workload and subsequently more control over the pace of work execution. Research from outside of healthcare, has identified the positive effects of planning behaviour (i.e., setting goals and priorities) and job autonomy on perceived control of time which, in turn, is positively related to job performance and job satisfaction. Further research should be conducted to examine the relationship between planning behaviour, autonomy, temporal enactments and staff outcomes within hospital settings, as well as research attempts to identify the potential *sweet spot* of optimal pace [1].

### Strengths and limitations

A strength of this study was the development of an initial psychometric profile for measuring temporality in the hospital setting, with its psychometric properties being assessed across four hospital sites in Australia. Another notable strength was the use of novel network analysis techniques to empirically examine the complex relationships between sociotemporal norms, safety attitudes and work-related wellbeing. As to limitations, the study was based on self-reports of staff and, as with all research of this kind, is reflective of the perceptions of the agents involved. We did not include patients’ self-reports or observational research, nor a measure of patient safety. The data was collected at one time point and therefore cannot identify any causal influence of temporality on staff wellbeing or safety attitudes; this would require longitudinal studies involving repeated sampling on the same set of study participants. The OTS-H also warrants further cross-validation of its factor structure, as the final 19 items were validated on the basis of results from our four included hospitals, and may not be generalisable to all hospital systems. Optimally, CFA should be randomly divided into subgroups (calibration and validation samples) to validate and verify the factor structure of the tool [53]. However, the current study was limited by the relatively modest sample size. Further, the main sources of missing data were for the OTS items, due to it being administered at the end of a very long survey. However, the demographic characteristics between included and excluded cases were not significantly different in terms of age, sex and years at hospital. Nevertheless, issues regarding the sample size, missing data as well as the inability to calculate a response rate limit generalisability of the results. Therefore, we would recommend that further research would be needed to verify the validity of the tool.

## Conclusions

As one of the first studies to empirically explore the relationship between temporal enactments, safety attitudes and work-related wellbeing, we found that pace occupies the central position in understanding the complex relationship amongst these variables. Our findings highlight the need for managerial interventions including promoting levels of trust in staff, and a shift in organisational policies aimed to give staff more control over the pace of their work as these are likely to have mediating or carryover effects on staff wellbeing and staff safety attitudes. Having tested the OTS-H, we offer it for future studies on this topic and highlight the adoption of novel network analysis approaches for the examination of a complex system of constructs as complementary to well-established methods, such as CFA and path analysis.

## Supplementary Information

**Additional file 1.** Table of survey item means and standard deviations.

## Data Availability

The datasets generated and analysed during the current study are not publicly available due to the data having been collected subject to the informed consent of the participants. Access to data is available from the corresponding author on reasonable request.

## Abbreviations

OTS: Organizational temporality scale
CFA: Confirmatory factor analysis
SFA: Safety attitudes questionnaire
JDS: Job diagnostic survey
MBI: Maslach burnout inventory
EM: Expectation maximisation
TLI: Tucker Lewis Index
CFI: Comparative Fit Index
RMSEA: Root Mean Square Error of Approximation
VIF: Variance inflation factor
