# Supplementary material for: “Time is of the essence”: relationship between hospital staff perceptions of time, safety attitudes and staff wellbeing
Source: BMC Health Serv Res. 2021 Nov 20;21:1256. doi: 10.1186/s12913-021-07275-6 (PMC8605531; doi:10.1186/s12913-021-07275-6)
Supplement: Supplementary file 1 — Additional file 1. Table of survey item means and standard deviations. [file 12913_2021_7275_MOESM1_ESM.docx]

**Additional File 1: Table of survey item means and standard deviations**

| **Factor** | **Abbreviated item name** | **Complete Item wording** | **Mean** | **SD** |
| --- | --- | --- | --- | --- |
| **Sociotemporal structures** | | | | |
| Flexibility | SETSTONE (R) | Set in stone | 4.29 | 1.34 |
|  | RIGID (R) | Rigid | 4.31 | 1.33 |
|  | FIXED (R) | Fixed | 4.15 | 1.33 |
|  | INFLEXIB (R) | Inflexible | 4.26 | 1.34 |
| Pace | FASTPACE* | Fast-paced | 4.43 | 1.44 |
|  | HURRIED | Hurried | 4.01 | 1.48 |
|  | RAPID | Rapid | 3.88 | 1.49 |
|  | QUICK | Quick | 4.11 | 1.43 |
|  | RACING | Racing | 3.60 | 1.53 |
| Linearity | ONEATIM* | Carried out one thing at a time | 3.24 | 1.38 |
|  | STRUCTUR | Structured | 3.84 | 1.28 |
|  | HAVORDER | Having a specific order | 3.88 | 1.26 |
|  | STPBYSTP | Carried out step by step | 3.88 | 1.26 |
| Scheduling | UNSCHED | Unscheduled | 3.31 | 1.46 |
|  | UNPLAN | Unplanned | 3.47 | 1.41 |
|  | ONTIME* | On time | 3.74 | 1.24 |
| Delay | BEHNDSCH | Behind schedule | 3.34 | 1.30 |
|  | RUNLATE | Running late | 3.26 | 1.29 |
|  | DELAYED | Delayed | 3.30 | 1.31 |
| Punctuality | PUNCTUAL | Punctual | 3.76 | 1.28 |
|  | PROMPT | Prompt | 3.93 | 1.24 |
| **Safety attitudes** | | | | |
| Teamwork climate | TMWK1 | Nurse input is well received in my work area | 3.72 | 0.81 |
|  | TMWK2 (R) | In my work area, it is difficult to speak up if I perceive a problem with patient care | 3.60 | 1.01 |
|  | TMWK3 | Disagreements in my work area are resolved appropriately (i.e. not who is right, but what is best for the patient) | 3.47 | 0.98 |
|  | TMWK4 | I have the support I need from other staff in my unit to care for patients | 3.87 | 0.91 |
|  | TMWK5 | It is easy for staff in this unit to ask questions when there is something that they do not understand | 3.96 | 0.89 |
|  | TMWK6 | The doctors and nurses here work together as a well-coordinated team | 3.67 | 0.91 |
| Safety climate | SAFE1 | I would feel safe being treated here as a patient | 3.73 | 0.89 |
|  | SAFE2 | Medical errors are handled appropriately in this unit | 3.73 | 0.79 |
|  | SAFE3 | I receive appropriate feedback about my performance | 3.39 | 1.07 |
|  | SAFE4 (R) | In my work area, it is difficult to discuss errors | 3.60 | 1.03 |
|  | SAFE5 | I am encouraged by my colleagues to report any patient safety concerns I may have | 3.84 | 0.82 |
|  | SAFE6 | The culture in my work area makes it easy to learn from the errors of others | 3.48 | 0.96 |
| **Burnout** | | | | |
| Emotional Exhaustion | EEXH1 | I feel emotionally drained from my work | 4.34 | 1.78 |
|  | EEXH2 | I feel tired when I get up in the morning and have to face another day on the job | 3.99 | 1.82 |
|  | EEXH3 | I feel burned out from my work | 3.80 | 1.80 |
|  | EEXH4 | I feel used up at the end of the workday | 4.25 | 1.83 |
|  | EEXH5 | Working all day is really a strain for me | 3.39 | 1.80 |
| Depersonalisation | DEPN1 | I have become less enthusiastic about my work | 3.64 | 1.95 |
|  | DEPN2 | I have become more cynical about whether my work contributes anything | 3.44 | 2.04 |
|  | DEPN3 | I have become less interested in my work since I started this job | 3.10 | 1.85 |
|  | DEPN4 | I just want to do my job and not be bothered | 3.24 | 1.91 |
|  | DEPN5 | I doubt the significance of my work | 2.89 | 1.83 |
| **Job Satisfaction** | | | | |
| Job Satisfaction | JBSN1 | All in all, I am satisfied with my job | 3.68 | 0.99 |
|  | JBSN2 (R) | In general, I don't like my job | 3.93 | 0.97 |
|  | JBSN3 | In general, I like working here | 3.91 | 0.86 |

Note. Analyses based on N=314. (R) Items reverse coded. *These items were dropped on the basis of CFA analyses
